# Systems-level molecular and immunological evidence identifies Th17/Treg modulation as a key mechanism of CRSJ’s neuroprotection in Parkinson’s disease

**DOI:** 10.3389/fnagi.2026.1764634

**Published:** 2026-02-18

**Authors:** Xun Li, XiYu Li, ShiYa Chen, Lin Wang, JinYan Xia, MeiLing Zheng, ChuTian Zhang, XiaoQian Chen, Jing Cai

**Affiliations:** 1 School of Integrative Chinese and Western Medicine, Fujian University of Traditional Chinese Medicine, Fuzhou, China; 2 The Third Affiliated People’s Hospital, Fujian University of Traditional Chinese Medicine, Fuzhou, China; 3 Department of Neurology, Hubei Provincial Hospital of Traditional Chinese Medicine, Wuhan, China; 4 The Second Affiliated People’s Hospital, Fujian University of Traditional Chinese Medicine, Fuzhou, China; 5 Clinical Research Center for Traditional Chinese Medicine on Glucolipid Metabolic Disorders of Fujian Province, Fuzhou, China

**Keywords:** Congrong Shujing Granules, systems-level, neuroprotection, Parkinson’s disease, Th17/Treg

## Abstract

**Background:**

Parkinson’s disease (PD) is a progressive neurodegenerative disorder in which neuroinflammation plays a central role. Congrong Shujing Granules (CRSJ), a traditional Chinese medicine formula, have shown clinical benefits in PD, yet their immunomodulatory mechanisms remain unclear.

**Methods:**

We investigated the effects of CRSJ on Th17/Treg immune balance. Liquid chromatography–tandem mass spectrometry (LC-MS/MS) was used to identify representative chemical constituents of CRSJ. Representative CRSJ compounds were characterized, and their binding affinities were evaluated by molecular docking and molecular dynamics simulations. An MPTP-induced PD mouse model was established and treated with CRSJ. Behavioral outcomes, dopaminergic neuroprotection, immune cell subsets, transcriptomic profiles, and cytokine networks were assessed using flow cytometry, RNA sequencing, multiplex assays, immunofluorescence, and Western blotting.

**Results:**

HPLC analysis identified 44 representative compounds in CRSJ spanning multiple chemical classes associated with immunomodulatory, neuroprotective, and antioxidant activities. Molecular-level prioritization of CRSJ-derived serum constituents highlighted paeoniflorin as a key Th17/Treg balance immunoregulatory candidate, exhibiting stable interactions with RORγt, Foxp3, and α-synuclein in molecular docking and molecular dynamics simulations. In an MPTP-induced Parkinson’s disease mouse model, CRSJ treatment dose-dependently improved motor performance, preserved dopaminergic neurons, and reduced striatal α-synuclein accumulation. Transcriptomic profiling revealed CRSJ-associated shifts toward regulatory immune programs, characterized by attenuation of Th17-related signatures and enhancement of Treg-associated pathways, accompanied by consistent modulation of the TGF-β/SMAD3 signaling axis. These molecular changes were supported by protein-level validation. CRSJ further alleviated neuroinflammation by promoting microglial M1/M2 polarization and partially normalizing dysregulated cytokine and chemokine profiles. Integrated immunological analyses demonstrated restoration of Th17/Treg balance and suppression of CX3CL1/CX3CR1–Th17 signaling, collectively supporting an immuno-neuroprotective profile of CRSJ in PD.

**Conclusion:**

CRSJ exerts neuroprotective effects in PD by restoring Th17/Treg homeostasis and suppressing neuroinflammatory pathways, supporting its potential as an immunomodulatory therapy.

## 1 Introduction

Parkinson’s disease (PD) is a prevalent neurodegenerative disorder primarily characterized by motor impairments such as resting tremor, muscular rigidity, bradykinesia, and postural instability. With the global population aging, the incidence of PD is steadily increasing, thereby imposing a growing social and economic burden and presenting a significant public health challenge worldwide (Su et al., 2025). Although the precise pathogenic mechanisms of PD are not yet fully elucidated, accumulating evidence suggests that neuroinflammation and immune dysregulation play significant roles in the disease’s onset and progression (Lind-Holm Mogensen et al., 2025).

Over the past decade, converging evidence has established a central role for T-cell dysregulation in PD. Postmortem studies reveal infiltration of CD4^+^ and CD8^+^ T lymphocytes into the PD brain, implicating adaptive immunity in neurodegeneration (Sun et al., 2024). Among CD4^+^ subsets, Th17 cells disrupt blood–brain barrier integrity through IL-17–mediated tight-junction breakdown, thereby promoting the entry of inflammatory mediators and autoreactive immune cells into the central nervous system (Liu et al., 2019). Patients with sporadic PD consistently exhibit increased frequencies of IL-17–producing CD4^+^ T cells in peripheral blood (Sommer et al., 2019), and α-synuclein–activated Th17 cells can directly induce dopaminergic neuron loss in experimental models (Clarke et al., 2025). By contrast, regulatory T cells (Tregs) restrain effector T-cell activity and maintain immune homeostasis (Wang L. et al., 2025). In MPTP-induced PD models, adoptive Treg transfer mitigates dopaminergic neurodegeneration and microglial activation while enhancing neurotrophic factor expression (Reynolds et al., 2007). Tregs can also cross the blood–brain barrier and acquire memory-like phenotypes within the brain parenchyma, where they dampen local inflammation and confer neuroprotection (Machhi et al., 2020). Together, these findings highlight the Th17/Treg axis as a mechanistic driver of PD pathogenesis and a promising target for therapeutic intervention (Chen et al., 2015; McGinley et al., 2020).

Traditional Chinese Medicine (TCM), with its multi-component and systems-level therapeutic framework, has shown growing potential in neuroprotection and is considered a promising source of novel interventions for PD (Hao et al., 2024; Li et al., 2024; Zhang et al., 2014). CRSJ, a classical TCM formula composed of Cistanche deserticola, processed Polygonatum, Salvia miltiorrhiza, Paeonia lactiflora, and Moutan cortex, is clinically used to alleviate PD symptoms (Chen S. Y. et al., 2020). In TCM, CRSJ is thought to tonify kidney function, promote blood circulation, resolve stasis, and clear internal heat. Pharmacological studies indicate that CRSJ enhances dopaminergic neuronal survival via Wnt/β-catenin–mediated upregulation of tyrosine hydroxylase (TH) (Xu et al., 2021). Its medicated serum contains major bioactive constituents—including echinacoside, paeoniflorin, salvianolic acid B, acteoside, and tanshinone IIa—suggesting synergistic anti-inflammatory actions (Zhang et al., 2025; Supplementary Table 1). Among them, paeoniflorin is well documented to modulate the Th17/Treg balance by suppressing Th17-related factors (RORγt, IL-17, IL-6) and promoting Treg markers (Foxp3, IL-10, TGF-β) (Wang C. et al., 2025). These findings support the potential immunomodulatory role of CRSJ in PD, although its precise therapeutic mechanisms remain to be fully elucidated.

This study investigates the neuroprotective effects of CRSJ in MPTP-induced PD mice, focusing on its capacity to modulate the Th17/Treg immune axis. By delineating how CRSJ influences the dynamic balance between Th17 and Treg cells, this work aims to provide mechanistic insight into the neuroimmune regulation exerted by traditional Chinese medicine formulas (Pu et al., 2025; Tian et al., 2025).

## 2 Materials and methods

### 2.1 Experimental drugs

The CRSJ formulation consisted of Cistanche deserticola (6 g), processed Polygonatum (12 g), Salvia miltiorrhiza (15 g), Paeonia lactiflora (12 g), and Moutan cortex (10 g) (Supplementary Table 2). All herbal materials were supplied by the Third Affiliated Hospital of Fujian University of Traditional Chinese Medicine. Each component was decocted twice in water for 1–2 h, and the combined filtrates were concentrated at 50–85°C, dried, and homogenized to obtain the crude extract. Extracts were mixed according to the prescribed ratios and processed into 12–40 mesh granules by dry granulation. Based on body surface area conversion (factor = 9.1), the mouse-equivalent dose for a 20 g animal was calculated as 3.71 g/kg. Three CRSJ dosage groups were therefore established: low (1.68 g/kg), medium (3.71 g/kg), and high (7.42 g/kg), corresponding to 0.5 × , 1 × , and 2 × the adult equivalent dose.

### 2.2 Liquid chromatography–tandem mass spectrometry analysis

A precisely weighed 0.2 g aliquot of CRSJ powder was extracted with 10 mL of 80% methanol containing grinding beads. After grinding for 5 min and vortexing for 10 min, the mixture was centrifuged at 13,000 rpm for 10 min, and the supernatant was collected for analysis. Mass spectrometry was performed on a Q Exactive instrument (Thermo Fisher Scientific, Shanghai, China) using electrospray ionization (ESI) in both positive and negative switching modes. Chromatographic separation employed an UltiMate 3000 RS HPLC system (Thermo Fisher Scientific, Shanghai, China) with an AQ-C18 column (150 × 2.1 mm, 1.8 μm; Welch) at a flow rate of 0.7 mL/min, using water with 0.1% formic acid as the mobile phase. The gradient elution program is provided in Supplementary Table 5. High-resolution spectra were preprocessed with Compound Discoverer 3.3 (Thermo Fisher Scientific) and searched against the mzCloud database. Compounds scoring above the threshold were initially selected and subsequently confirmed by MS^2^ and MS^2^ spectral matching.

### 2.3 Molecular dock

Two-dimensional ligand structures were retrieved from PubChem, and their three-dimensional conformations were generated in ChemOffice and saved as mol2 files. High-resolution crystal structures of target proteins were obtained from the RCSB PDB. All protein structures were prepared in PyMOL by removing water molecules and other heteroatoms. Docking was performed using AutoDock Vina (version 1.5.6). Protein and ligand structures were processed by adding hydrogens, assigning charges, and defining rotatable bonds. Grid box coordinates were set according to the predicted binding pocket. The best binding pose was selected based on docking affinity scores. Protein–ligand interactions were visualized using Discovery Studio 2019. Binding affinity < −5.0 kcal/mol was considered indicative of good binding, whereas < −7.0 kcal/mol indicated strong binding. Lower binding energy reflects higher affinity and greater conformational stability.

### 2.4 Molecular dynamics

Molecular dynamics (MD) simulations were performed using GROMACS 2022 to evaluate the stability of protein–ligand complexes. The CHARMM36 force field was applied to proteins, and ligand parameters were generated using CGenFF. Complexes were solvated in a TIP3P water box with a 1.2 nm margin and neutralized with counterions. Long-range electrostatics were treated with the particle-mesh Ewald (PME) method, and the Verlet cutoff scheme was used. After energy minimization, NVT and NPT equilibration were performed for 200 ps each with position restraints on protein heavy atoms. Production MD simulations were run for 100 ns at 310 K and 1 bar. RMSD and RMSF analyses were performed to assess structural stability and residue flexibility (Li X. et al., 2025).

### 2.5 Animal studies

Male C57BL/6J mice (8 weeks, 20 ± 2 g) were obtained from the Laboratory Animal Center of Fujian University of Traditional Chinese Medicine and housed in SPF conditions [license: SYXK (Min) 2023-0004]. All procedures were approved by the Institutional Animal Care and Use Committee (FJTCM IACUC 0024037). After 1-week acclimation, 88 mice received intraperitoneal injections of MPTP (30 mg/kg/day) for 7 days to induce PD-like symptoms, while 12 mice served as untreated controls. Based on behavioral scoring (Supplementary Table 3), 60 successfully modeled mice were randomized into five groups: model, CRSJ-L (low-dose Congrong Shujing Granules, 1.68 g/kg), CRSJ-M (medium-dose Congrong Shujing Granules, 3.71 g/kg), and CRSJ-H (high-dose Congrong Shujing Granules, 7.42 g/kg), and Madopar (0.05 g/kg). Treatments were administered once daily by oral gavage for 14 days. Body weight was recorded weekly, and doses were adjusted accordingly. At the study endpoint, mice were anesthetized with pentobarbital sodium (150 mg/kg), and striatum and spleen were collected for analysis.

### 2.6 Behavioral assessments

Mice were habituated to the testing room for 1 h before each assessment. Behavioral tests—including the wire hang test, pole test, and open-field test—were conducted on day 9 after MPTP induction and on days 7 and 14 of treatment. Detailed scoring criteria are provided in Supplementary Tables 4, 5. In the pole test, mice were placed head-up at the top of a gauze-wrapped wooden pole (50 × 1 cm) and the time to descend was recorded. In the open-field test, mice were placed in the center of a 40 × 40 × 25 cm arena divided into 16 squares, and locomotor activity was monitored for 5 min using the TOP SCAN Super Maze 3.0 system. The apparatus was cleaned with 75% ethanol between trials.

### 2.7 Immunohistochemistry

Brain tissues were collected from treated mice and fixed in 4% paraformaldehyde, followed by dehydration, clearing, paraffin embedding, and sectioning into 5 μm slices. After deparaffinization and rehydration, antigen retrieval was performed in citrate buffer (pH 6.0) at 95–100°C for 15 min. Sections were then blocked with 5% goat serum for 30 min at room temperature. Subsequently, they were incubated overnight at 4°C with an anti–tyrosine hydroxylase primary antibody (Proteintech, 25859-1-AP). The next day, sections were incubated for 1 h with an HRP-conjugated goat anti-mouse/rabbit IgG polymer secondary antibody (Boster, SA1020). DAB chromogenic development was carried out using a commercial kit (Boster, AR1022) for 1–3 min until optimal staining was achieved, followed by dehydration and mounting. Images were acquired under a light microscope at 100 × magnification. For quantification, 3–5 sections per mouse were analyzed, and three randomly selected fields per section were imaged. TH-positive neurons in the substantia nigra were quantified using ImageJ to obtain the mean neuronal count for each animal.

### 2.8 Flow cytometry

Fresh spleens were gently dissociated through a 70-μm cell strainer to obtain single-cell suspensions, followed by red blood cell lysis. Cells were stimulated with PMA (Elabscience, No. E-CK-A091) for 5 h at 37°C in the presence of a protein transport inhibitor. After stimulation, cells were fixed and permeabilized using the FoxP3/Transcription Factor Staining Buffer Kit (LianKe, No. IC001). For Treg staining, cells were incubated with FITC-conjugated anti-CD4 (Elabscience, No. E-AB-F1353C), APC-conjugated anti-CD25 (Elabscience, No. E-AB-F1102E), and PE-conjugated anti-Foxp3 (Elabscience, No. E-AB-F1238D). For Th17 staining, cells were labeled with FITC-anti-CD4 and APC-anti-IL-17A antibodies (Elabscience, No. E-AB-F1199E). After washing and filtration to remove bubbles and aggregates, samples were analyzed on an LSRFortessa™ flow cytometer (BD Biosciences, New Jersey, United States). Based on established gating strategies, CD4^+^CD25^+^Foxp3^+^ cells were defined as Tregs, and CD4^+^IL-17A^+^ cells were identified as Th17 cells.

### 2.9 Immunofluorescence

Paraffin-embedded brain tissue sections were deparaffinized, rehydrated, and subjected to antigen retrieval. Sections were permeabilized with 1% Triton X-100 (Servicebio, No. G1204), washed with 0.1% PBS-T, and blocked with 10% goat serum at 37°C for 30 min. Primary antibodies against Foxp3 (Servicebio, No. GB112325-50), CX3CL1 (Proteintech, No. 60339), RORγt (Affinity, No. DF3196), IBA1 (Servicebio, No. GB12105), CD206 (Servicebio, No. GB113497), CD86 (Servicebio, No. GB150054), and IL-17 (Servicebio, No. GB11110) were applied and incubated overnight at 4°C. The next day, fluorescently labeled secondary antibodies (Servicebio, No. GB22303, GB27301, or GB28301) were added and incubated for 1 h at room temperature in the dark. Slides were mounted using DAPI-containing mounting medium (Servicebio, No. G1401). Fluorescence images were captured under identical exposure settings using a fluorescence microscope, and positive signals were quantified using Fiji software.

### 2.10 Western blot

Brain tissue samples were lysed using a protein extraction buffer (Servicebio, No. G2002), and protein concentrations were determined using the BCA assay. Equal amounts of protein were separated by SDS-PAGE and transferred onto PVDF membranes (Servicebio, No. G6045). Membranes were blocked with 5% non-fat milk at room temperature for 1.5 h, followed by overnight incubation at 4°C with primary antibodies against RORγt (Affinity, No. DF3196), CX3CR1 (Proteintech, No.60339), α-synuclein (Servicebio, No. GB11773), Foxp3 (Servicebio, No. GB112325), IL-17A (No. GB11110), Arg1 (Servicebio, No. GB11285), iNOS (Servicebio, No. GB153965), TGF-β (Servicebio, No. GB111876), smad3 (Servicebio, No. GB150085), and β-actin (Servicebio, No. GB15003). Membranes were then incubated with appropriate secondary antibodies for 1 h (Servicebio, No. GB23303, GB23301). Protein bands were visualized using a Bio-Rad chemiluminescence imaging system, and relative expression levels were quantified using AIWBwell™ analysis software.

### 2.11 Luminex liquid suspension chip assay

Serum samples were fully thawed at room temperature and diluted 1:4 before loading into 96-well plates for multiplex cytokine analysis using the Luminex^®^ 200 system (Luminex Corporation, Texas, United States). A 31-plex cytokine/chemokine panel (Bio-Rad, Bio-Plex Pro™ Mouse Chemokine Panel 31-Plex, No. 12009159) was used according to the manufacturer’s instructions. Samples and standards were incubated with magnetic beads for 1.5 h at room temperature in the dark with gentle shaking, followed by a series of wash steps and incubation with detection antibodies and streptavidin–PE. All serum samples were measured in duplicate. Quantification was based on bead classification and fluorescence intensity, and data acquisition and analysis were performed using Bio-Plex Manager™ software.

### 2.12 RNA sequencing and transcriptomic analysis

Two mice were randomly selected from each group for collection of bilateral striatum tissues. For RNA-seq, tissues from each mouse were processed independently, with each sample treated as one biological replicate. Total RNA was extracted using the MJzol Animal RNA Isolation Kit (Majorbio) and further purified with the RNAClean XP Kit (Beckman Coulter) and RNase-Free DNase Set (QIAGEN). rRNA-depleted, strand-specific libraries were constructed from each sample.

Sequencing was performed on the Illumina NovaSeq 6000 platform to generate 150-bp paired-end reads, yielding approximately 30 million reads per sample. Raw reads were subjected to quality control and trimming using TrimGalore (v0.6.x) and seqtk (v1.0). Clean reads were aligned to the mouse reference genome (GRCm39) using HISAT2 (v2.2.x), and alignment files were processed with SAMtools (v1.9) and Sambamba (v0.6.4). Transcript quantification was performed using StringTie (v1.3.3b) to obtain gene-level expression matrices. Differentially expressed genes (DEGs) were identified using edgeR (v3.2.0) with thresholds of Q-value ≤ 0.05 and | log*2* fold change| ≥ 1. To investigate Th17/Treg-related immunoregulatory programs, single-sample gene set enrichment–like analyses were performed using curated Th17/Treg, IL-6–STAT3, and TGFβ–SMAD pathway signatures. Pathway scores and immune marker expression patterns were visualized using heatmaps. Given the limited sample size, pathway-level trends and coordinated transcriptional patterns were emphasized.

### 2.13 Antibody

The antibodies used in the IHC, IF, FCM, and WB are listed in Supplementary Tables 6–8.

### 2.14 Statistical analysis

All data are presented as mean ± standard deviation (mean ± SD). Statistical analyses were conducted using IBM SPSS Statistics 26.0, and graphical representations were generated with GraphPad Prism 9.2. Data normality and homogeneity of variance were assessed using the Shapiro–Wilk test, respectively. For data meeting the assumptions of normal distribution and homogeneity of variance, one-way analysis of variance followed by Tukey’s *post-hoc* test was used for multiple group comparisons. When the assumption of homogeneity of variance was violated, the Games–Howell *post-hoc* test was applied. Non-normally distributed data were analyzed using non-parametric tests. The significance level was set at α, = 0.05, with *p* < 0.05 considered statistically significant.

## 3 Results

### 3.1 Multi-class bioactive compounds in CRSJ support coordinated regulation of immune balance, neuroinflammation, and oxidative stress

**FIGURE 1:**
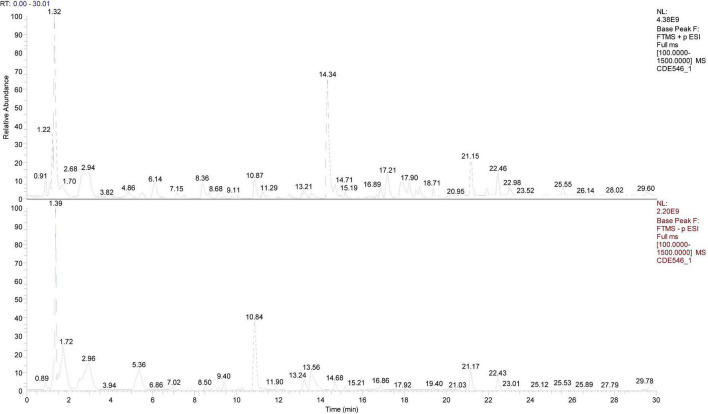
LC–MS/MS total ion chromatogram of CRSJ. Column 1 shows the total ion current in the positive ion mode, and column 2 shows the total ion current in the negative ion mode. CRSJ, Congrong Shujing Granules; LC–MS/MS, liquid chromatography-tandem mass spectrometry.

Liquid chromatography–tandem mass spectrometry (LC–MS/MS) analysis of the CRSJ aqueous extract identified 44 representative compounds derived from its constituent herbs (Figure 1 and Table 1). Based on prior pharmacological evidence, these compounds were functionally associated with immunomodulatory and neuroprotective activities through partially overlapping pathways. Specifically, paeoniflorin and its analogs (albiflorin and oxypaeoniflorin), together with verbascoside, genistein, and paeonol, have been linked to regulation of the Th17/Treg axis via suppression of RORγt/IL-17 signaling and enhancement of Foxp3- and IL-10–related pathways. Salvianolic acid A and B, rosmarinic acid, danshensu, tanshinone IIA, and cryptotanshinone have been associated with attenuation of microglial activation, oxidative stress, and α-synuclein aggregation (Table 1). Additional phenolic compounds, including tyrosol, caffeic acid, ferulic acid, gallic acid, ellagic acid, and isomangiferin, are implicated in mitochondrial and redox homeostasis (Table 1). Oligosaccharides such as stachyose, raffinose, and nystose have been reported to contribute to peripheral immune regulation and Treg induction (Table 1). Collectively, the identified compounds span multiple chemical classes and suggest a multi-component profile consistent with coordinated regulation of immune balance, neuroinflammation, and oxidative stress.

**TABLE 1:** Representative bioactive compounds of CRSJ.

Herb	Identified compound	Chemical class	RT (min)	Representative biological relevance
Cistanche deserticola	Betaine	Alkaloid/osmolyte	1.67	Anti-inflammatory, methyl donor, immune modulation
Cistanche deserticola	Verbascoside	Phenylethanoid glycoside	11.906	Anti-inflammatory, antioxidant, Th17/Treg regulation
Cistanche deserticola	Tubuloside A	Phenylethanoid glycoside	11.657	Neuroprotection, anti-oxidative stress
Cistanche deserticola	Aucubin	Iridoid glycoside	6.232	Anti-inflammatory, neuroprotective
Cistanche deserticola	Tyrosol	Phenolic alcohol	10.169	Antioxidant, mitochondrial protection
Cistanche deserticola	Caffeic acid	Phenolic acid	10.264	Anti-inflammatory, ROS scavenging
Cistanche deserticola	Ferulic acid	Phenolic acid	11.903	Neuroprotection, anti-oxidative stress
Cistanche deserticola	Verbenalin	Iridoid glycoside	10.473	Anti-inflammatory, immune regulation
Salvia miltiorrhiza	Danshensu	Phenolic acid	7.031	Anti-inflammatory, microcirculatory protection
Salvia miltiorrhiza	Salvianolic acid A	Polyphenolic acid	14.069	Anti-neuroinflammation, antioxidant
Salvia miltiorrhiza	Salvianolic acid B	Polyphenolic acid	13.596	Neuroprotection, α-syn aggregation inhibition
Salvia miltiorrhiza	Lithospermic acid	Polyphenolic acid	13.137	Anti-inflammatory, vascular protection
Salvia miltiorrhiza	Cryptotanshinone	Diterpenoid quinone	18.726	Immunomodulation, Th17 inhibition
Salvia miltiorrhiza	Tanshinone IIA	Diterpenoid quinone	19.66	Anti-neuroinflammation, microglial regulation
Salvia miltiorrhiza	Dihydrotanshinone I	Diterpenoid quinone	17.661	Anti-inflammatory, neuroprotective
Salvia miltiorrhiza	Rosmarinic acid	Polyphenolic acid	13.231	Antioxidant, immune modulation
Paeonia lactiflora	Paeoniflorin	Monoterpene glycoside	10.865	Th17/Treg rebalancing, neuroimmune regulation
Paeonia lactiflora	Albiflorin	Monoterpene glycoside	10.086	Anti-inflammatory, neuroprotection
Paeonia lactiflora	Oxypaeoniflorin	Monoterpene glycoside	9.401	Immunomodulation
Paeonia lactiflora	Benzoylpaeoniflorin	Monoterpene glycoside	14.699	Anti-inflammatory
Paeonia lactiflora	Methyl gallate	Phenolic ester	8.88	Antioxidant, anti-inflammatory
Moutan ortex/Paeonia lactiflora	Gallic acid	Phenolic acid	5.361	Antioxidant, immune regulation
Moutan cortex	Paeonol	Phenolic ketone	14.334	Anti-neuroinflammation, microglial inhibition
Moutan cortex	Ellagic acid	Polyphenol	13.823	Anti-inflammatory, antioxidant
Polygonatum sibiricum	Genistein	Isoflavone	18.761	Th17 inhibition, Treg promotion
Polygonatum sibiricum	Hesperidin	Flavonoid glycoside	12.91	Anti-inflammatory, neuroprotection
Polygonatum sibiricum	Stachyose	Oligosaccharide	1.482	Gut–immune–brain axis modulation
Polygonatum sibiricum	Raffinose	Oligosaccharide	1.505	Immune homeostasis, prebiotic effect
Polygonatum sibiricum	Nystose	Oligosaccharide	2.231	Treg induction, immune tolerance
Polygonatum sibiricum	Isomangiferin	Xanthone glycoside	1.83	Antioxidant, neuroprotective

Compounds were identified based on accurate mass, retention time or published MS databases.

### 3.2 Molecular-level evidence supports paeoniflorin as a Th17/Treg immunomodulator hub modulating Th17/Treg balance among CRSJ-derived serum constituents

**FIGURE 2:**
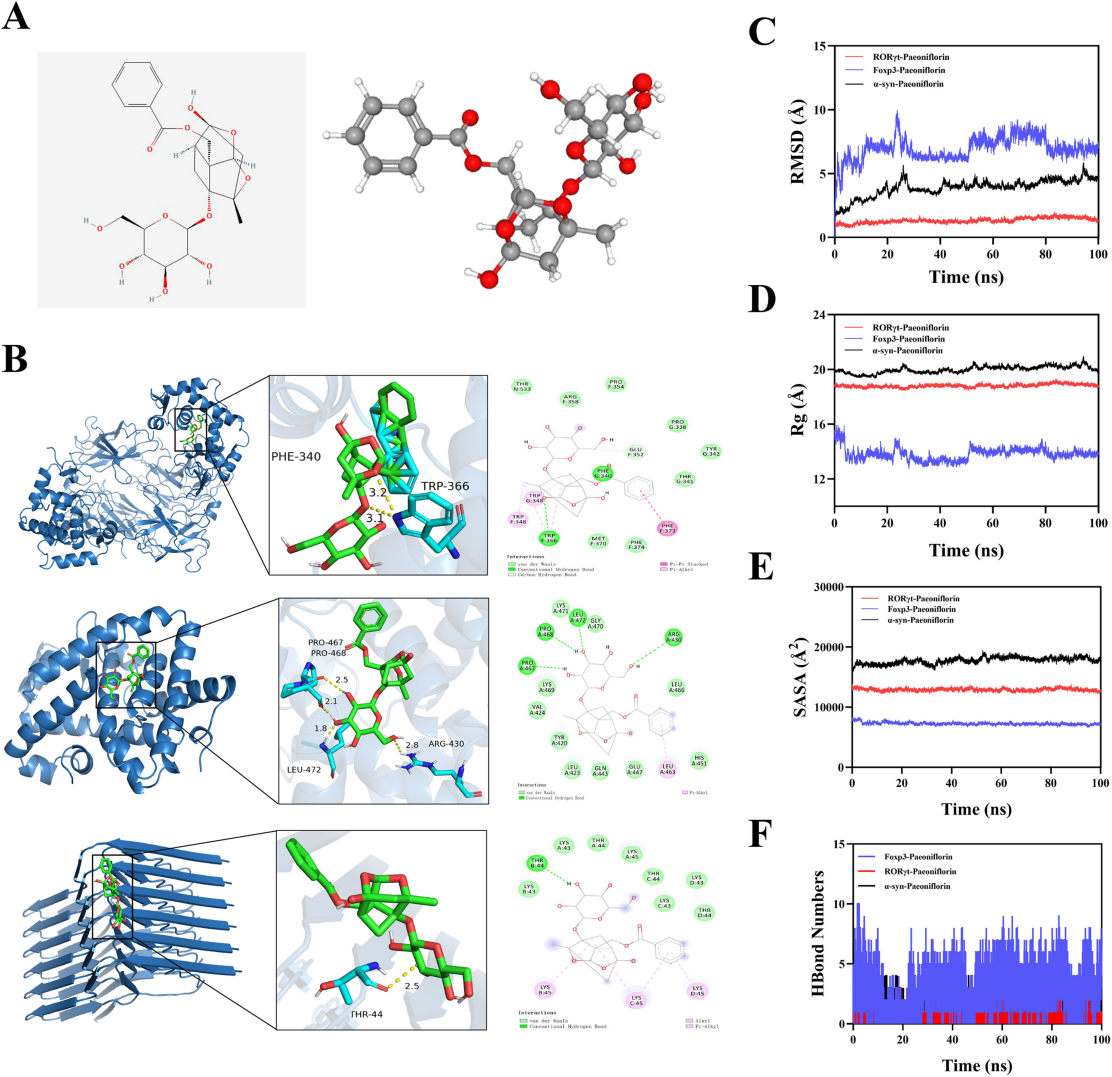
Molecular docking and molecular dynamics simulations of paeoniflorin–protein complexes. **(A)** Chemical structure and three-dimensional conformation of paeoniflorin. **(B)** Predicted binding poses of paeoniflorin within the active sites of Foxp3, RORγt, and α-synuclein, obtained by molecular docking analysis. **(C)** RMSD profiles of paeoniflorin–protein complexes during the MD simulation. **(D)** Rg, reflecting the compactness and structural stability of each complex. **(E)** SASA of the complexes throughout the simulation period, indicating dynamic changes in protein–ligand exposure to solvent. **(F)** Time-dependent hydrogen bond number between paeoniflorin and target proteins, used to evaluate binding stability. RMSD, Root mean square deviation; SASA, solvent-accessible surface area; Rg, Radius of Gyration.

CRSJ-medicated serum contains multiple bioactive constituents, including echinacoside, paeoniflorin, salvianolic acid B, acteoside, and tanshinone IIA, consistent with a cooperative anti-inflammatory profile. Among these, paeoniflorin (PF) has been extensively implicated in Th17/Treg regulation across inflammatory and autoimmune models, primarily through suppression of Th17-associated signaling and enhancement of Treg-related pathways (Figures 2A). Based on this established immunomodulatory relevance, PF was prioritized for molecular docking and molecular dynamics analyses with Foxp3, RORγt, and α-synuclein. Paeoniflorin exhibited favorable binding affinities toward all three targets, with relatively stronger interactions observed for RORγt (Figures 2B and Supplementary Table 9). Stable protein–ligand associations were supported by hydrogen bonding and hydrophobic interactions, and molecular dynamics simulations confirmed conformational stability of the complexes, particularly for the RORγt–paeoniflorin interaction (Figures 2C–F). Rather than assuming uniform bioactivity across all detected constituents, we integrated serum exposure, prior pharmacological evidence, and pathway relevance to prioritize functionally plausible components. Within this framework, paeoniflorin emerges as a key immunoregulatory node linked to Th17/Treg modulation, while other constituents likely provide complementary anti-inflammatory, antioxidant, and neuroprotective support. This systems-level chemical architecture aligns with the multi-target pharmacology of traditional formulations and offers a mechanistic basis for the immuno-neuroprotective effects of CRSJ.

### 3.3 CRSJ improves motor function and neuromuscular performance in MPTP-induced PD mice

**FIGURE 3:**
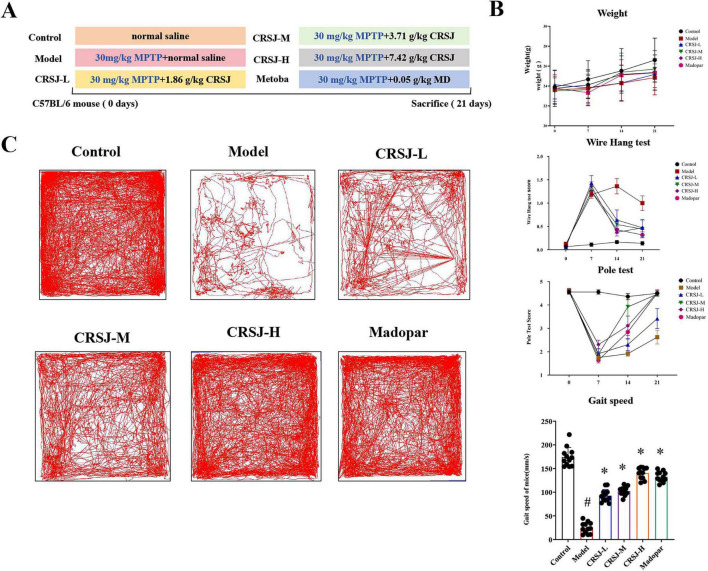
CRSJ ameliorates motor deficits in MPTP-induced PD mice. **(A)** Experimental design and timeline. **(B)** Body weight, hanging test scores, pole test performance, and gait velocity in the nesting test before and after CRSJ treatment (*n* = 12). **(C)** Summary of locomotor trajectories of mice in the open field test (*n* = 12). *P* < 0.05 vs. Model group; *P < 0.05 vs. Control group. CRSJ-L, low-dose Congrong Shujing Granules; CRSJ-M, medium-dose Congrong Shujing Granules; and CRSJ-H, high-dose Congrong Shujing Granules.

To assess CRSJ’s therapeutic efficacy, we established an MPTP-induced PD mouse model and evaluated motor performance (Figure 3A). Baseline body weight and motor performance were comparable across all groups prior to MPTP administration (Figure 3B). Following MPTP induction, PD model mice exhibited significant impairments in body weight gain, neuromuscular strength, and motor coordination, as reflected by altered wire hang and pole test performance, confirming successful model establishment (Figures 3B,C). CRSJ treatment dose-dependently improved body weight and motor function after 1 and 2 weeks of intervention, with the mid- and high-dose groups showing the most pronounced recovery, comparable to Madopar. Although motor performance did not fully return to baseline, all CRSJ-treated groups performed significantly better than the untreated model group, indicating that CRSJ effectively ameliorates MPTP-induced motor dysfunction. Overall, these findings indicate that CRSJ effectively alleviates MPTP-induced motor dysfunction and enhances neuromuscular performance in PD mice.

### 3.4 CRSJ preserves dopaminergic neurons and reduces α-Syn accumulation in PD mice

**FIGURE 4:**
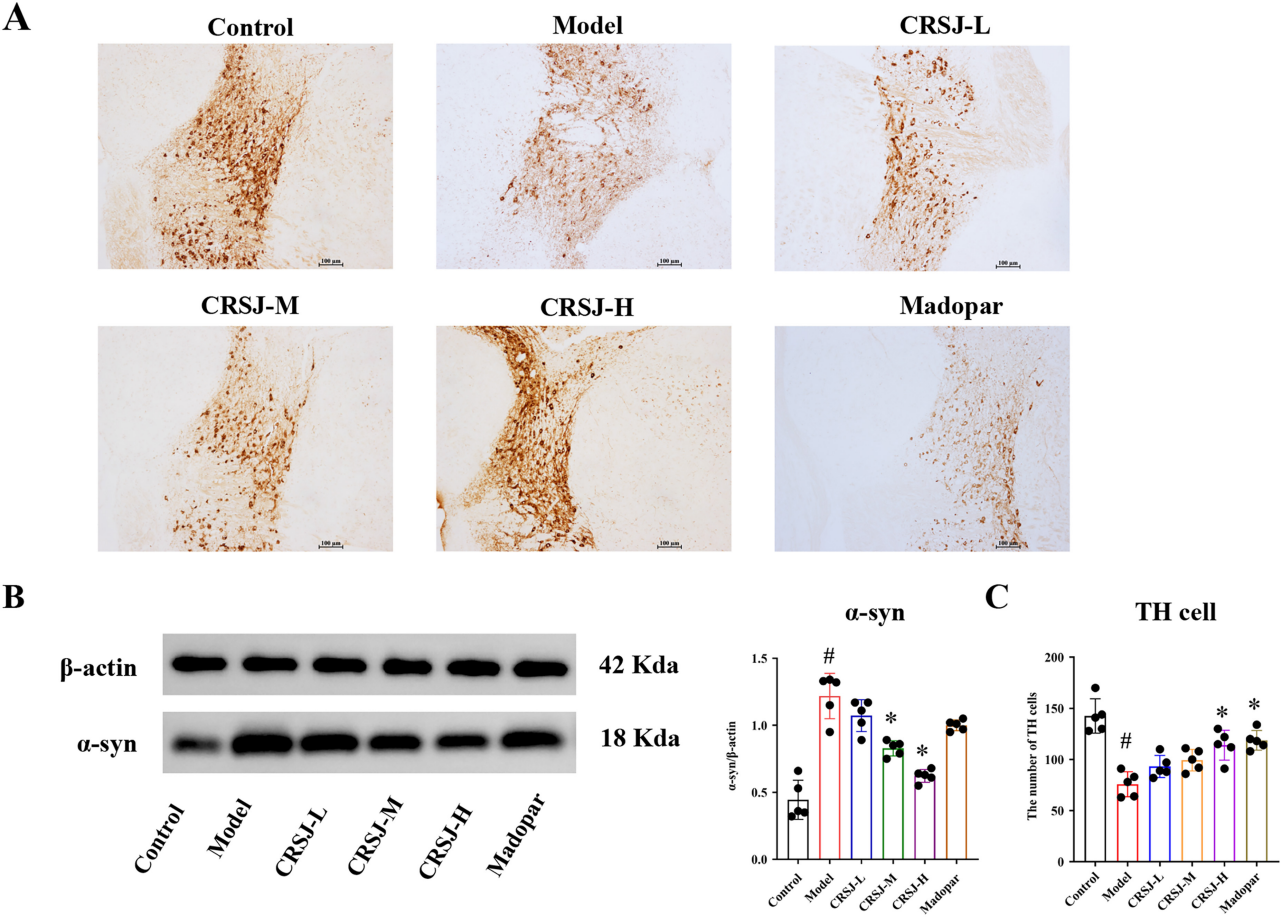
CRSJ alleviates neuropathological alterations in the substantia nigra and striatum of PD mice. **(A)** Tyrosine hydroxylase (TH) immunoreactivity in the substantia nigra (*n* = 5). **(B)** Western blot and quantitative analysis of α-synuclein expression in the striatum (*n* = 5). **(C)** Quantification of TH-positive neurons in the substantia nigra (*n* = 5). **#***P* < 0.05 vs. Model group; *P < 0.05 vs. Control group. CRSJ-L, low-dose Congrong Shujing Granules; CRSJ-M, medium-dose Congrong Shujing Granules; CRSJ-H, high-dose Congrong Shujing Granules.

To assess the neuroprotective effects of CRSJ, we examined dopaminergic neuron survival in the substantia nigra and α-syn protein expression in the striatum of MPTP-induced PD mice. TH immunohistochemistry revealed a marked reduction of TH-positive neurons in the model group (*P* < 0.05), confirming dopaminergic degeneration (Figures 4A,C). CRSJ treatment significantly preserved TH-positive cells in a dose-dependent manner, with mid- and high-dose groups showing the strongest protection, comparable to the Madopar control (Figure 4C). Western blot analysis further demonstrated elevated α-syn expression in the striatum of model mice relative to controls (*P* < 0.05), consistent with pathological aggregation. CRSJ intervention significantly reduced α-syn levels, particularly in the mid- and high-dose groups, whereas the low-dose group showed a non-significant downward trend (Figure 4B). Together, these findings indicate that CRSJ suppresses abnormal α-syn accumulation and preserves dopaminergic neurons, supporting its neuroprotective potential in PD.

### 3.5 Transcriptomic profiling reveals that CRSJ restores Th17/Treg balance associated with modulation of the TGF-β/SMAD3 signaling axis

**FIGURE 5:**
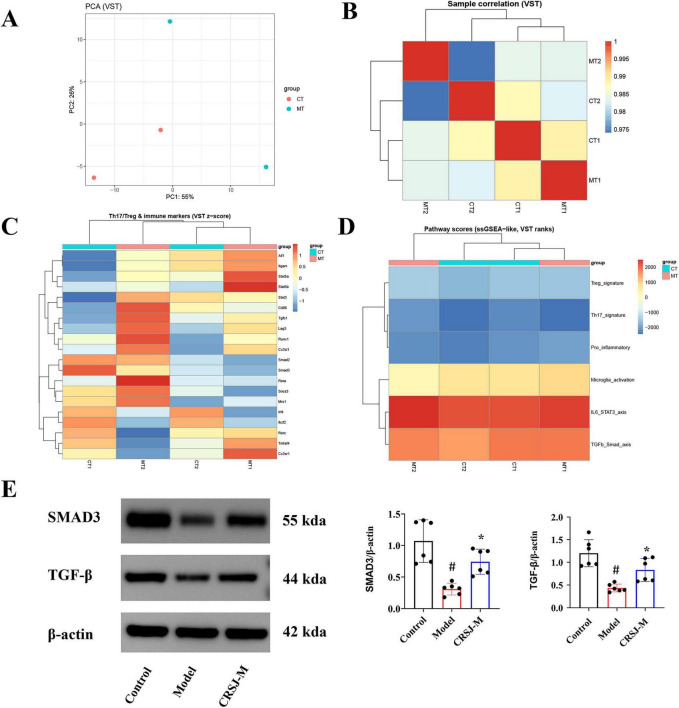
Exploratory transcriptomic analysis reveals CRSJ-associated Th17/Treg immune-related expression patterns. **(A)** PCA based on VST–normalized expression values. **(B)** Sample-to-sample correlation heatmap generated from VST-normalized expression data. **(C)** Hierarchical clustering heatmap of selected Th17/Treg-associated and immune regulatory genes based on VST z-score–normalized expression values. **(D)** Heatmap of ssGSEA-like pathway activity scores estimated from VST-ranked gene expression data. **(E)** Western blot validation and quantitative analysis of TGF-β signaling pathway–related proteins (*n* = 6). #*P* < 0.05 vs. model group; *P < 0.05 vs. control group. CRSJ-M, medium-dose Congrong Shujing Granules; CT, control group; MT, model group. PCA, Principal component analysis; VST, variance-stabilized transformation; SsGSEA, Single-sample GSEA.

To characterize the molecular correlates of CRSJ-mediated immunomodulation in Parkinson’s disease, transcriptomic profiling was performed on striatal tissues from PD mice treated with mid-dose CRSJ. Principal component analysis (PCA) of variance-stabilized expression values showed clear separation between the model (MT) and CRSJ-treated (CT) groups along the first principal component, accounting for 55% of total variance (Figure 5A). Consistently, sample correlation analysis demonstrated high intra-group similarity and distinct inter-group clustering, indicating treatment-associated transcriptional differences with minimal technical variability (Figure 5B). Analysis of Th17/Treg-related immune markers revealed a coordinated shift toward a regulatory transcriptional profile following CRSJ treatment. Treg-associated components, including Tgfb1, Smad2/3, and Stat5 family members, were relatively upregulated, whereas Th17-associated regulators (Rorc, Rora, Stat3) and immune activation markers were attenuated (Figure 5C), indicating restoration of Th17/Treg-associated transcriptional balance. Pathway-level assessment using single-sample GSEA–like scoring further demonstrated coordinated pathway activity shifts, including suppression of Th17-related programs and the IL-6–STAT3 axis, accompanied by enhancement of Treg-associated signatures and the TGF-β–SMAD signaling pathway, together with reduced pro-inflammatory and microglial activation signatures in CRSJ-treated mice (Figure 5D). Among these pathways, TGF-β signaling emerged as a consistently altered feature across multiple analytical layers. Consistent with the transcriptomic results, Western blot analysis confirmed increased protein expression of TGF-β and SMAD3 in the striatum of CRSJ-treated mice compared with the PD model group (*P* < 0.05) (Figure 5E). Collectively, these data indicate that CRSJ treatment is associated with restoration of Th17/Treg-related immune programs, accompanied by consistent modulation of the TGF-β/SMAD3 signaling axis.

### 3.6 CRSJ shifts microglial polarization from a pro-inflammatory M1 phenotype toward an anti-inflammatory M2 state in PD mice

**FIGURE 6:**
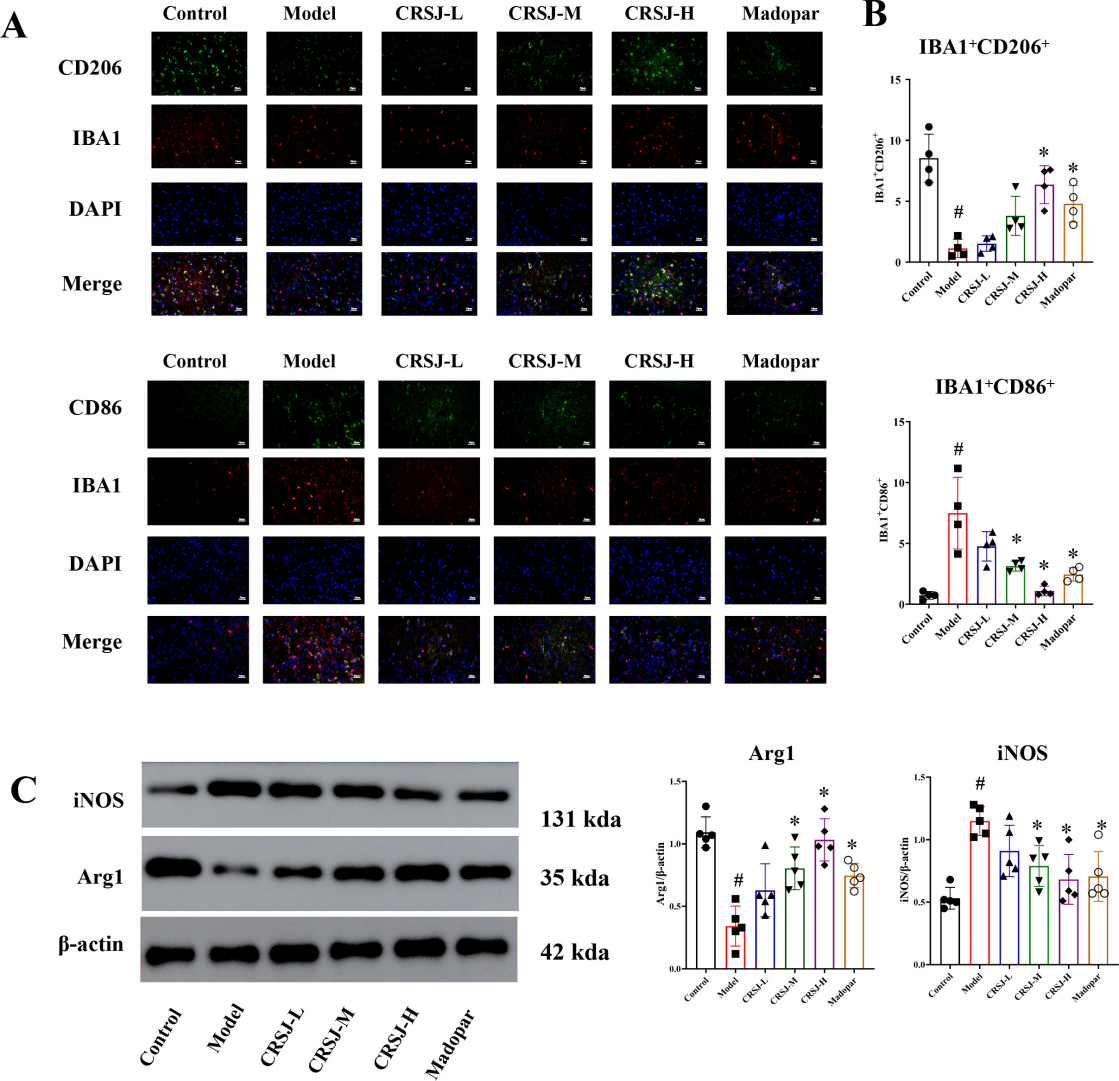
CRSJ associated modulation of M1/M2 immune-related expression markers. **(A)** Immunofluorescent staining of IBA1^+^CD86^+^ and IBA1^+^CD206^+^ expression (*n* = 4). **(B)** quantification of IBA1^+^CD86^+^ and IBA1^+^CD206^+^ expression (*n* = 4). **(C)** Western blot and quantitative analysis of iNOS and Arg1 proteins (*n* = 5). **#***P* < 0.05 vs. Model group; *P < 0.05 vs. Control group. CRSJ-L, low-dose Congrong Shujing Granules; CRSJ-M, medium-dose Congrong Shujing Granules; CRSJ-H, high-dose Congrong Shujing Granules.

Given the established links between Th17/Treg imbalance, dysregulated TGF-β/SMAD3 signaling, and microglial activation, we next examined whether CRSJ modulates microglial M1/M2 polarization in a PD mouse model. Western blot analysis showed that PD model mice exhibited significantly increased expression of the M1 marker inducible nitric oxide synthase (iNOS) and reduced expression of the M2 marker arginase-1 (Arg1) compared with normal controls (*P* < 0.05) (Figure 6C). CRSJ treatment markedly reversed this polarization shift, with decreased iNOS and increased Arg1 levels across medium-, and high-dose groups (*P* < 0.05) (Figure 6C). Immunofluorescence double staining further confirmed these effects at the cellular level. The proportion of IBA1^+^CD86^+^ microglia was significantly increased in the model group, whereas IBA1^+^CD206^+^ cells were reduced (*P* < 0.05) (Figures 6A,B). CRSJ treatment significantly decreased IBA1^+^CD86^+^ co-expression in CRSJ-M and CRSJ-H, but not in the low-dose group (*P* > 0.05) (Figure 6A). In contrast, IBA1^+^CD206^+^ polarization was significantly enhanced only in the high-dose group, with no significant differences observed in the low- or medium-dose groups compared with the model group (*P* > 0.05) (Figures 6A,B). The Madopar group showed partial improvement. Together, these findings indicate that CRSJ shifts microglial polarization from a pro-inflammatory M1 phenotype toward an anti-inflammatory M2 state in the PD brain.

### 3.7 CRSJ attenuates cytokine and chemokine dysregulation in PD mice

**FIGURE 7:**
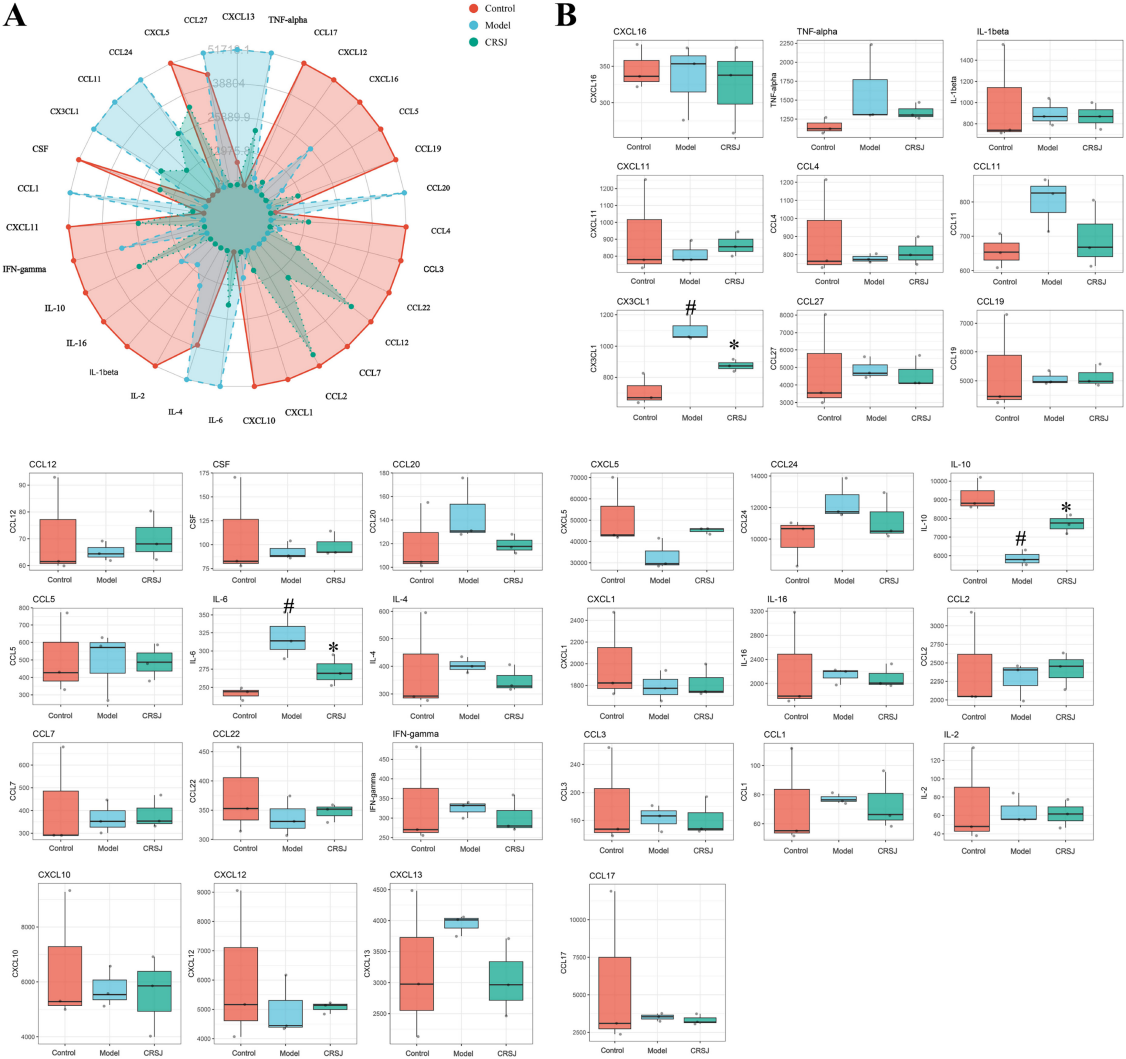
Serum cytokine expression profiles. **(A)** Immuno-radar plot illustrating the expression levels of 31 serum cytokines across groups (*n* = 3). **(B)** Boxplots showing the distribution of individual cytokines (*n* = 3). #*P* < 0.05 vs. Model group; *P < 0.05 vs. Control group. CRSJ, medium-dose Congrong Shujing Granules.

Thirty one cytokines and chemokines was quantified to characterize systemic immune alterations. The Model group showed broad pro-inflammatory activation, with notable increases in neuroimmune chemokines (CX3CL1, CXCL10, CXCL12, CCL4) and interleukins such as IL-1β, IL-16, and IL-4, confirming heightened microglial and peripheral immune activity. CRSJ treatment partially normalized these abnormalities, reducing CX3CL1, CXCL10, CXCL12, TNF-α, IL-1β, and recruitment-related chemokines (CCL2, CCL3, CCL22), while markers such as CCL7, CCL12, and CCL20 remained largely unchanged (Figures 7A,B). Consistent with these trends, IL-6 was significantly elevated and IL-10 reduced in PD mice (*P* < 0.05), indicating Th17/Treg imbalance; CRSJ effectively reversed both changes (*P* < 0.05) (Figure 7B). Together, these results demonstrate that CRSJ attenuates PD-associated cytokine dysregulation and restores key neuroimmune regulatory pathways.

### 3.8 CRSJ restores Th17/Treg immune balance by modulating RORγt and Foxp3 expression

**FIGURE 8:**
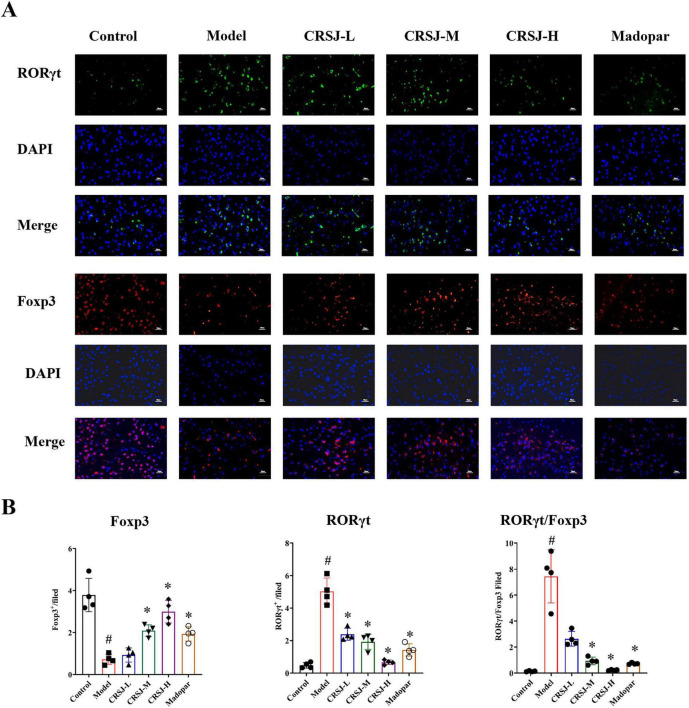
Th17/Treg transcription factors distribute. **(A)** Immunofluorescent staining of RORγt and Foxp3 expression (*n* = 4). **(B)** quantification of RORγt and Foxp3 expression (*n* = 4). **#***P* < 0.05 vs. Model group; *P < 0.05 vs. Control group. CRSJ-L, low-dose Congrong Shujing Granules; CRSJ-M, medium-dose Congrong Shujing Granules; CRSJ-H; high-dose Congrong Shujing Granules.

**FIGURE 9:**
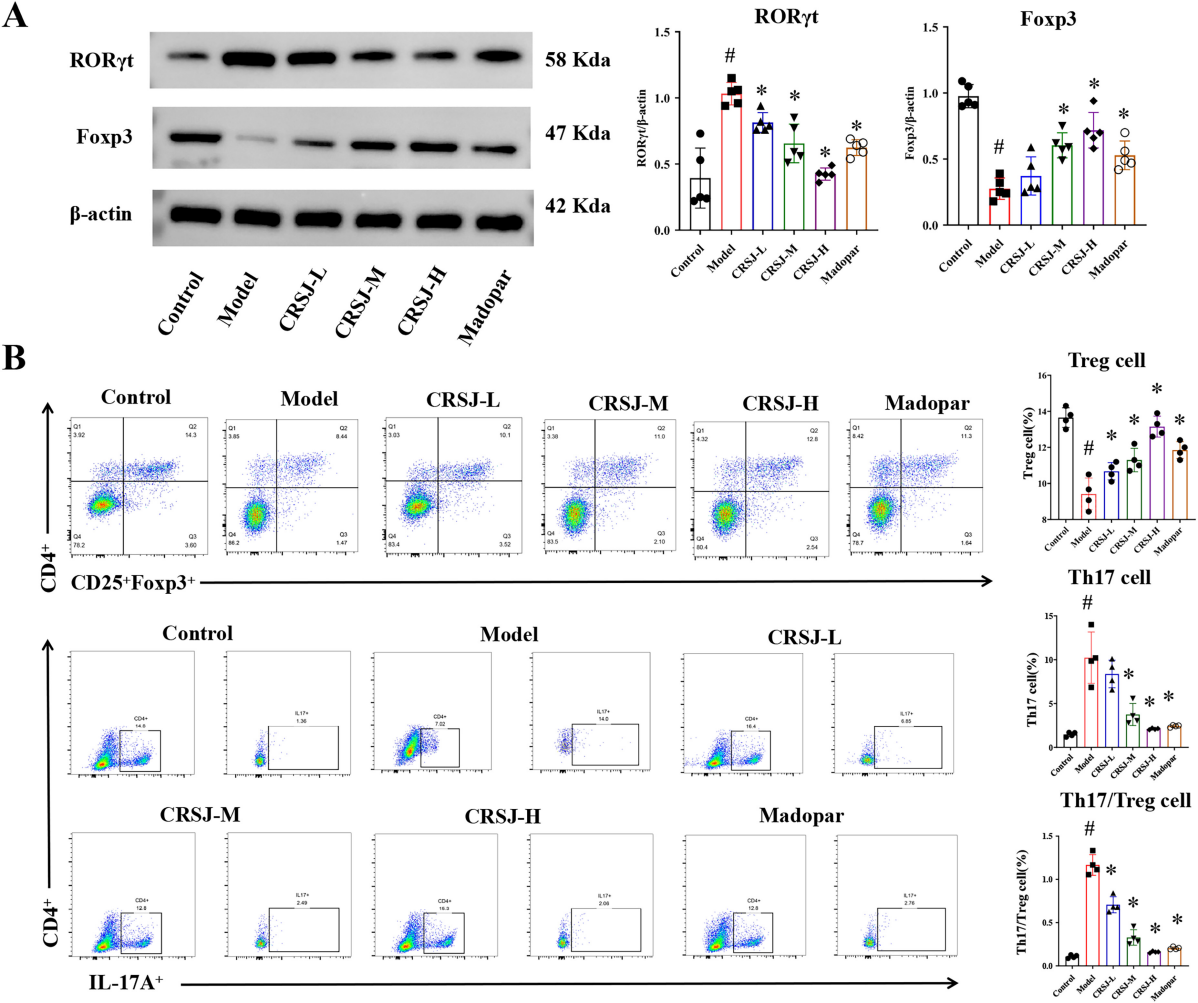
Quantification of Th17 and Treg cells and their transcription factor expression. **(A)** Western blot and quantitative analysis of Foxp3 and RORγt proteins (*n* = 5). **(B)** Flow cytometric profiling and quantification of Th17 and Treg cell populations (*n* = 4). ^#^*P* < 0.05 vs. Model group; *P < 0.05 vs. Control group. CRSJ-L, low-dose Congrong Shujing Granules; CRSJ-M, medium-dose Congrong Shujing Granules; CRSJ-H, high-dose Congrong Shujing Granules.

To assess CRSJ’s immunomodulatory effects, we examined Th17/Treg-associated transcription factors and cell populations. In PD mice, RORγt expression was increased and Foxp3 was decreased in the mesencephalic region, indicating Th17-skewed immune differentiation (Figure 8A). CRSJ treatment significantly reduced RORγt expression across all doses, whereas Foxp3 expression and the RORγt/Foxp3 ratio were significantly corrected only in the medium- and high-dose groups but not in the low-dose group compared with the model group (*P* > 0.05), with similar effects observed in the Madopar group (Figure 8B). Flow cytometry further confirmed systemic immune imbalance: model mice displayed increased Th17 (CD4^+^IL-17A^+^) and decreased Treg (CD4^+^CD25^+^Foxp3^+^) populations, resulting in a disrupted Th17/Treg ratio (Figure 9B). Mid- and high-dose CRSJ effectively normalized these proportions (*P* < 0.05). Correspondingly, Western blot analysis showed striatum of upregulated RORγt and downregulated Foxp3 in model mice, both of which were significantly corrected by CRSJ-M, CRSJ-H, and Madopar (Figure 9A). Together with cytokine profiling and immunofluorescence data, these results demonstrate that CRSJ restores Th17/Treg homeostasis by modulating transcriptional programs and T-cell differentiation at multiple regulatory levels.

### 3.9 CRSJ suppresses CX3CL1/CX3CR1 signaling and Th17 activation in PD mice

**FIGURE 10:**
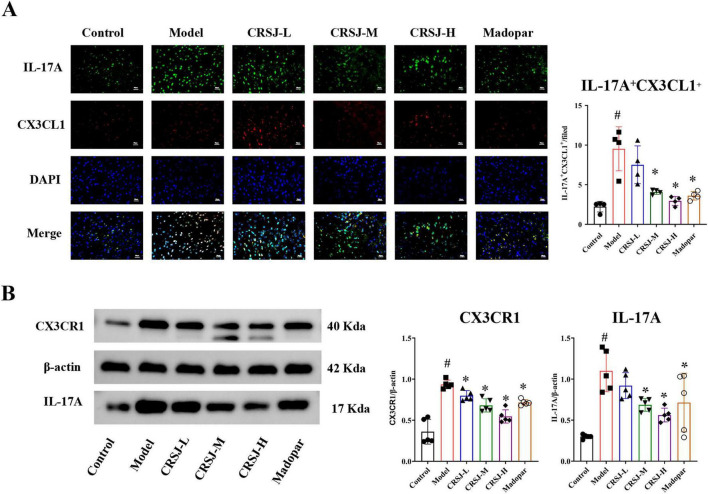
Expression of CX3CL1 and Th17 Cells in PD mice. **(A)** Co-localization of CX3CL1 and IL-17A in immunofluorescence images, along with quantitative analysis and serum CX3CL1 levels (*n* = 4). **(B)** Western blot and quantitative analysis of CX3CL1 and IL-17A (*n* = 5). **#***P* < 0.05 vs. Model group; *P < 0.05 vs. Control group. CRSJ-L, low-dose Congrong Shujing Granules; CRSJ-M, medium-dose Congrong Shujing Granules; CRSJ-H, high-dose Congrong Shujing Granules.

To examine whether CRSJ regulates chemokine CX3CL1 and Th17-cell–related immune responses in PD mice, we quantified CX3CL1 expression and key Th17-associated indicators. Among these, CX3CL1 was significantly elevated in the Model group compared with controls (*P* < 0.05), and was markedly reduced following CRSJ-M and CRSJ-H treatment (*P* < 0.05) (Figure 7B). To assess downstream mediators, striatal expression of CX3CR1—the cognate receptor of CX3CL1—and IL-17A, a key Th17 effector molecule, was examined. Western blotting revealed significant upregulation of both CX3CR1 and IL-17A in the Model group, which were significantly suppressed by CRSJ-M, CRSJ-H, and Madopar treatments (*P* < 0.05); IL-17A reductions in the CRSJ-L group did not reach statistical significance (*P* > 0.05) (Figure 10A). Dual immunofluorescence co-localization analysis further demonstrated increased CX3CL1–IL-17A co-expression in the Model group (*P* < 0.05), which was significantly attenuated by all CRSJ doses and Madopar (*P* < 0.05) (Figure 10B). Collectively, these results show that CRSJ effectively modulates CX3CL1/CX3CR1 signaling and Th17-related activity, thereby contributing to restoration of Th17/Treg balance and exerting neuroprotective effects in PD.

## 4 Discussion

PD is the second most common neurodegenerative disorder worldwide, with a rapidly increasing prevalence driven by global population aging (Luo et al., 2024). PD is characterized by progressive dopaminergic neuron loss, reduced TH expression, and pathological α-syn aggregation, features faithfully reproduced in our MPTP-induced model (Nagatsu, 2024; Praveen et al., 2025). CRSJ significantly improved motor coordination and locomotor performance, indicating functional rescue at the behavioral level. Mechanistically, CRSJ preserved TH-positive neurons in the substantia nigra and reduced α-syn accumulation in the striatum. Such dual engagement of immune-regulatory pathways and protein aggregation processes supports the disease-modifying potential of CRSJ rather than merely symptomatic relief.

Th17 and Treg cells drive opposing immunological outcomes in neurodegeneration. Th17 cells, regulated by RORγt, secrete IL-17A and IL-6, disrupt blood–brain barrier integrity, and exacerbate dopaminergic neurotoxicity (Chen J. et al., 2020; Jiang et al., 2025; Liu et al., 2022; Shi et al., 2022; Zheng and Luo, 2025). Conversely, Foxp3^+^ Treg cells secrete IL-10 and TGF-β, attenuating microglial activation and promoting neuronal survival (Huang et al., 2020; Sun and Guo, 2025; Xie et al., 2024). Our data show that PD mice exhibit a pronounced Th17/Treg imbalance, reflected by increased Th17 cell numbers, elevated RORγt and IL-17A expression, and reduced Foxp3 and IL-10 signals—consistent with clinical and preclinical observations that Treg exhaustion and Th17 overactivation contribute to PD progression (Li et al., 2023). CRSJ reversed these abnormalities at transcriptional, protein, and cellular levels. By suppressing RORγt and restoring Foxp3, CRSJ shifts immune programming toward a regulatory phenotype, thereby reducing neuroinflammation and neuronal vulnerability. This supports the concept that Th17/Treg rebalancing is a crucial mechanism of CRSJ’s therapeutic action.

Transcriptomic analyses suggested that CRSJ treatment was associated with coordinated changes in immune- and neuroinflammation-related networks in the striatum, rather than widespread gene-level changes. Although few genes met stringent differential expression criteria, pathway- and module-level analyses consistently highlighted immunoregulatory signaling, with the TGF-β pathway emerging as a dominant feature. Single-sample GSEA–like pathway scoring and immune marker profiling further demonstrated suppression of Th17-associated transcriptional programs accompanied by enhancement of Treg-related signaling following CRSJ treatment. Collectively, these findings highlight the TGF-β/SMAD3 axis as a recurrently modulated pathway, characterized by upregulation of TGF-β/SMAD signaling components and concomitant attenuation of the IL-6–STAT3 pathway, a key driver of Th17 differentiation (Cheng et al., 2025; Park and Ciofani, 2025; Xu Lou et al., 2025). Consistent with these transcriptomic findings, increased TGF-β and SMAD3 protein expression was confirmed in the striatum, supporting a model in which CRSJ confers neuroprotection by selectively reinforcing Treg-mediated immunoregulatory networks while limiting Th17-driven neuroinflammation and microglial overactivation.

Accumulating evidence indicates that Th17/Treg imbalance is closely linked to maladaptive microglial activation in Parkinson’s disease (Roodveldt et al., 2024). Consistent with this framework, our data show that CRSJ shifts microglial polarization from a pro-inflammatory M1 phenotype toward an anti-inflammatory M2 state, as evidenced by reduced iNOS, increased Arg1 expression, and reciprocal changes in IBA1^+^CD86^+^ and IBA1^+^CD206^+^ microglia. Th17 effector signaling, particularly via the IL-17/IL-17R axis, promotes microglial activation and neuroinflammatory amplification (Li T. et al., 2025), whereas regulatory T cells constrain CNS innate immune responses (Jie et al., 2026). Together with emerging transcriptomic evidence that immune-intrinsic programs reshape Th17 differentiation, these findings support a model in which restoration of Th17/Treg balance is closely associated with normalization of microglial states (Sun et al., 2026). These cellular alterations parallel transcriptomic signatures of restored Th17/Treg balance and attenuation of Th17-associated inflammatory programs, suggesting that CRSJ modulates microglial activation primarily through upstream immune differentiation rather than direct inhibition of terminal inflammatory mediators. Although changes in TGF-β/SMAD3 signaling were observed, this pathway likely represents one component of a broader, multi-pathway immunoregulatory network. Accordingly, the neuroprotective effects of CRSJ are best interpreted as arising from integrated modulation of immune programs that collectively restrain chronic neuroinflammation and support dopaminergic neuron resilience.

CX3CL1 emerged as a pivotal chemokine altered in the PD model. Although CX3CL1 can exert context-dependent neuroprotective effects (Chu et al., 2025; Gutiérrez et al., 2025), many studies report early stage elevation and later decline during PD progression (Gutiérrez et al., 2025; Iemmolo et al., 2023; Li et al., 2022; Qu et al., 2023), reflecting dynamic microglial–neuronal interactions. Importantly, CX3CL1 is tightly linked to Th17 biology: IL-17A induces CX3CL1 expression, and CX3CL1/CX3CR1 signaling promotes Th17 chemotaxis and polarization (Akiyama et al., 2025; Yang et al., 2025). We observed significant increases in CX3CL1, CX3CR1, and Th17 markers in PD mice, supporting a cooperative inflammatory amplification loop (Li X. et al., 2025). CRSJ markedly suppressed CX3CL1/CX3CR1 expression and reduced IL-17A levels, indicating that CRSJ may disrupt this chemotactic–inflammatory circuit. By breaking this feedback loop, CRSJ limits Th17 recruitment and activation, thereby reducing inflammatory burden on vulnerable dopaminergic neurons. These findings highlight CX3CL1 as an immune–chemotactic target modulated by CRSJ and underscore its potential relevance in PD immunopathology.

CRSJ, as a traditional multi-herb formulation, exhibits therapeutic potential in PD. LC–MS analysis identified 44 representative compounds in the CRSJ aqueous extract spanning multiple chemical classes with established immunomodulatory and neuroprotective relevance. By integrating serum exposure, prior pharmacological evidence, and pathway involvement, we prioritized functionally plausible constituents rather than assuming uniform bioactivity. Recent studies support that paeoniflorin/total glucosides of peony and verbascoside-containing preparations can modulate Th17/Treg homeostasis (Zhao et al., 2025; Zheng et al., 2020), consistent with suppression of Th17-skewing inflammatory programs and enhancement of regulatory outputs (e.g., IL-10/Foxp3), while paeonol-containing regimens have likewise been reported to normalize Th17/Treg-related immune imbalance in inflammatory settings (Shi et al., 2021). In parallel, salvianolic acids (Akhtar et al., 2025; Bi et al., 2025), tanshinones (Zeng et al., 2024), and related phenolics have been linked to attenuation of microglial activation, oxidative stress, and α-synuclein pathology (Thi Lai et al., 2024; Yang et al., 2024). Consistent with this prioritization, molecular docking and molecular dynamics simulations identified paeoniflorin as a key immunoregulatory constituent, exhibiting stable binding to Foxp3, RORγt, and α-synuclein, with the strongest affinity toward RORγt. Collectively, these findings position paeoniflorin as a functionally prioritized immunoregulatory component, supported by complementary anti-inflammatory and neuroprotective constituents, providing a mechanistic basis for the multi-target immuno-neuroprotective effects of CRSJ.

While this study provides convergent evidence that CRSJ modulates Th17/Treg differentiation, suppresses chemokine-driven neuroinflammation, and attenuates α-syn pathology, several limitations should be acknowledged. First, although microglial polarization was systematically characterized, additional immune components, including B-cell subsets and α-syn–reactive antibody-producing cells, were not comprehensively examined and may represent further layers of CRSJ-mediated immunoregulation. Future work using multiparameter flow cytometry, CyTOF, or single-cell RNA sequencing will allow cell type–resolved mapping of CRSJ-induced immune responses in the central nervous system, together with detailed pharmacokinetic and bioavailability profiling of prioritized CRSJ constituents. Second, part of the immunological evidence was generated in an exploratory context, with transcriptomic analyses based on two biological replicates per group and flow cytometry performed in four mice per group. Although sufficient to identify consistent immune signatures and pathway-level alterations supported by independent validation, these sample sizes limit statistical power and sensitivity to inter-individual variability. Finally, reliance on a single MPTP-induced mouse model and the absence of human immune or clinical data constrain direct translational inference, warranting validation in additional PD models and human-derived samples.

In summary, this study identifies CRSJ as a conceptually distinct immunomodulatory strategy for Parkinson’s disease. Unlike existing approaches that target single inflammatory mediators or discrete immune cell subsets, CRSJ exerts coordinated, multi-target regulation across Th17/Treg differentiation, chemokine signaling, microglial activation, and α-syn–associated pathology. Such network-level immune reprogramming may be particularly advantageous in the multifactorial and dynamic inflammatory milieu of PD. Nevertheless, the exploratory nature of the transcriptomic analyses, limited sample sizes, reliance on a single toxin-induced model, and absence of human immune data constrain direct translational inference. Future studies incorporating larger cohorts, complementary disease models, and human-derived samples will be essential to validate and refine the therapeutic potential of CRSJ.

## 5 Conclusion

Collectively, this study provides convergent evidence that CRSJ treatment is associated with improved motor performance, preservation of dopaminergic neurons, and attenuation of α-synuclein accumulation in a Parkinson’s disease mouse model. These benefits are accompanied by coordinated modulation of immune-related processes, including restoration of Th17/Treg-associated immune balance, suppression of CX3CL1/CX3CR1–related inflammatory signaling, and consistent regulation of the TGF-β–SMAD3 pathway, with downstream normalization of microglial activation and overall reduction of neuroinflammation. Our findings support a multi-component, synergistic mode of action, in which paeoniflorin acts in concert with other active constituents to modulate interconnected immune, inflammatory, and proteostatic pathways. Although limited by sample size and the absence of human validation, this work provides a mechanistic foundation for the further development of CRSJ as a disease-modifying, immunoregulatory strategy for Parkinson’s disease.

## Data Availability

The datasets presented in this study can be found in online repositories. The names of the repository/repositories and accession number(s) can be found in this article/Supplementary material.

## References

[B1] Akhtar A. Singh P. Admane N. Grover A. (2025). Salvianolic acid B prevents the amyloid transformation of A53T mutant of α-synuclein. *Biophys. Chem.* 318:107379. 10.1016/j.bpc.2024.107379 39693815

[B2] Akiyama M. Wakasugi S. Yoshimoto K. Saito K. Ishigaki S. Inukai R. (2025). CX3CR1+ age-associated CD4^+^ T cells contribute to synovial inflammation in late-onset rheumatoid arthritis. *Inflamm. Regen.* 45:4. 10.1186/s41232-025-00367-4 39910629 PMC11800492

[B3] Bi S. Liu S. Zhu K. Gao D. Chen L. Yu C. (2025). Preclinical and experimental evidence of salvianolic acid B in the treatment of neurological diseases. *Front. Pharmacol.* 16:1606146. 10.3389/fphar.2025.1606146 40657643 PMC12245778

[B4] Chen J. Liu X. Zhong Y. (2020). Interleukin-17A: The key cytokine in neurodegenerative diseases. *Front. Aging Neurosci.* 12:566922. 10.3389/fnagi.2020.566922 33132897 PMC7550684

[B5] Chen S. Y. Xiao S. J. Lin Y. N. Li X. Y. Xu Q. Yang S. S. (2020). Clinical efficacy and transcriptomic analysis of congrong shujing granules () in patients with Parkinson’s disease and syndrome of shen (Kidney) essence deficiency. *Chin. J. Integr. Med.* 26 412–419. 10.1007/s11655-020-3080-0 32291608

[B6] Chen Y. Qi B. Xu W. Ma B. Li L. Chen Q. (2015). Clinical correlation of peripheral CD4^+^-cell sub-sets, their imbalance and Parkinson’s disease. *Mol. Med. Rep.* 12 6105–6111. 10.3892/mmr.2015.4136 26239429

[B7] Cheng H. Nan F. Ji N. Ma X. Zhang J. Liang H. (2025). Regulatory T cell therapy promotes TGF-β and IL-6-dependent pro-inflammatory Th17 cell generation by reducing IL-2. *Nat. Commun.* 16:7644. 10.1038/s41467-025-62628-7 40818959 PMC12357911

[B8] Chu Y. Harms A. S. Boehringer A. Kordower J. H. (2025). Decreased neuronal and increased endothelial fractalkine expression are associated with neuroinflammation in Parkinson’s disease and related disorders. *Front. Cell. Neurosci.* 19:1557645. 10.3389/fncel.2025.1557645 40842561 PMC12364955

[B9] Clarke J. R. Bacelar T. S. Fernandes G. G. Silva R. C. D. Antonio L. S. Queiroz M. (2025). Abatacept inhibits Th17 differentiation and mitigates α-synuclein-induced dopaminergic dysfunction in mice. *Mol. Psychiatry* 30 547–555. 10.1038/s41380-024-02618-1 39152331

[B10] Gutiérrez I. L. Martín-Hernández D. MacDowell K. S. García-Bueno B. Caso J. R. Leza J. C. (2025). CX3CL1 regulation of gliosis in neuroinflammatory and neuroprotective processes. *Int. J. Mol. Sci.* 26:959. 10.3390/ijms26030959 39940727 PMC11817243

[B11] Hao Y. Li J. Dan L. Wu X. Xiao X. Yang H. (2024). Chinese medicine as a therapeutic option for pulmonary fibrosis: Clinical efficacies and underlying mechanisms. *J. Ethnopharmacol.* 318(Pt A):116836. 10.1016/j.jep.2023.116836 37406748

[B12] Huang Y. Liu Z. Cao B. B. Qiu Y. H. Peng Y. P. (2020). Treg cells attenuate neuroinflammation and protect neurons in a mouse model of Parkinson’s disease. *J. Neuroimmune Pharmacol.* 15 224–237. 10.1007/s11481-019-09888-5 31802419

[B13] Iemmolo M. Ghersi G. Bivona G. (2023). The cytokine CX3CL1 and ADAMs/MMPs in concerted cross-talk influencing neurodegenerative diseases. *Int. J. Mol. Sci.* 24:8026. 10.3390/ijms24098026 37175729 PMC10179166

[B14] Jiang Z. Huang H. Chen Y. Xie H. Lu Y. Ge Y. (2025). The role of the immune system in Parkinson’s disease pathogenesis: A focus on Th17 cells - A systematic review and meta-analysis. *J. Neuroimmunol.* 398:578484. 10.1016/j.jneuroim.2024.578484 39577101

[B15] Jie J. Yao X. Deng H. Zhou Y. Jiang X. Dai X. (2026). Regulatory T cells in neurological disorders and tissue regeneration: Mechanisms of action and therapeutic potentials. *Neural Regen. Res.* 21 1277–1291. 10.4103/NRR.NRR-D-24-01363 40536993 PMC12407513

[B16] Li J. Zhao J. Chen L. Gao H. Zhang J. Wang D. (2023). α-Synuclein induces Th17 differentiation and impairs the function and stability of Tregs by promoting RORC transcription in Parkinson’s disease. *Brain Behav. Immun.* 108 32–44. 10.1016/j.bbi.2022.10.023 36343753

[B17] Li M. Wang H. Bai Y. Xiong F. Wu S. Bi Q. (2024). Pharmacodynamical research of extracts and compounds in traditional Chinese medicines for Parkinson’s disease. *Fitoterapia* 177:106086. 10.1016/j.fitote.2024.106086 38897243

[B18] Li Q. Han X. Dong M. Bai L. Zhang W. Liu W. (2025). FDA-approved secukinumab alleviates glial activation and immune cell infiltration in MPTP-induced mouse model of Parkinson’s disease. *Inflammation* 48 3314–3325. 10.1007/s10753-025-02267-8 40011292 PMC12596411

[B19] Li T. Qiu T. Jiang F. Cai H. Le W. (2025). Microglia in the crosstalk between peripheral and central nervous systems in Parkinson’s disease. *Transl. Neurodegener.* 14:66. 10.1186/s40035-025-00531-3 41372772 PMC12696919

[B20] Li X. Cai J. Xia J. Zheng M. (2025). *Multicomponent Mendelian Randomization and Machine Learning Studies of Potential Drug Targets for Neurodegenerative Diseases.* Berlin: Springer Science and Business Media.

[B21] Li Y. Yang Y. Zhao A. Luo N. Niu M. Kang W. (2022). Parkinson’s disease peripheral immune biomarker profile: A multicentre, cross-sectional and longitudinal study. *J. Neuroinflammation* 19:116. 10.1186/s12974-022-02481-3 35610646 PMC9131564

[B22] Lind-Holm Mogensen F. Seibler P. Grünewald A. Michelucci A. (2025). Microglial dynamics and neuroinflammation in prodromal and early Parkinson’s disease. *J. Neuroinflammation* 22:136. 10.1186/s12974-025-03462-y 40399949 PMC12096518

[B23] Liu S. Y. Qiao H. W. Song T. B. Liu X. L. Yao Y. X. Zhao C. S. (2022). Brain microglia activation and peripheral adaptive immunity in Parkinson’s disease: A multimodal PET study. *J. Neuroinflammation* 19:209. 10.1186/s12974-022-02574-z 36038917 PMC9422161

[B24] Liu Z. Qiu A. W. Huang Y. Yang Y. Chen J. N. Gu T. T. (2019). IL-17A exacerbates neuroinflammation and neurodegeneration by activating microglia in rodent models of Parkinson’s disease. *Brain Behav. Immun.* 81 630–645. 10.1016/j.bbi.2019.07.026 31351185

[B25] Luo Y. Qiao L. Li M. Wen X. Zhang W. Li X. (2024). Global, regional, national epidemiology and trends of Parkinson’s disease from 1990 to 2021: Findings from the Global Burden of Disease Study 2021. *Front. Aging Neurosci.* 16:1498756. 10.3389/fnagi.2024.1498756 39868382 PMC11757241

[B26] Machhi J. Kevadiya B. D. Muhammad I. K. Herskovitz J. Olson K. E. Mosley R. L. (2020). Harnessing regulatory T cell neuroprotective activities for treatment of neurodegenerative disorders. *Mol. Neurodegener.* 15:32. 10.1186/s13024-020-00375-7 32503641 PMC7275301

[B27] McGinley A. M. Sutton C. E. Edwards S. C. Leane C. M. DeCourcey J. Teijeiro A. (2020). Interleukin-17A serves a priming role in autoimmunity by recruiting IL-1β-producing myeloid cells that promote pathogenic T cells. *Immunity* 52 342–356.e6. 10.1016/j.immuni.2020.01.002 32023490

[B28] Nagatsu T. (2024). Catecholamines and Parkinson’s disease: Tyrosine hydroxylase (TH) over tetrahydrobiopterin (BH4) and GTP cyclohydrolase I (GCH1) to cytokines, neuromelanin, and gene therapy: A historical overview. *J. Neural Transm.* 131 617–630. 10.1007/s00702-023-02673-y 37638996

[B29] Park E. Ciofani M. (2025). Th17 cell pathogenicity in autoimmune disease. *Exp. Mol. Med.* 57 1913–1927. 10.1038/s12276-025-01535-9 40887501 PMC12508148

[B30] Praveen A. Dougnon G. Matsui H. (2025). Exploring α-Syn’s functions through ablation models: Physiological and pathological implications. *Cell. Mol. Neurobiol.* 45:44. 10.1007/s10571-025-01560-2 40389720 PMC12089638

[B31] Pu W. Pan Y. Yang K. Gao J. Tian F. Song J. (2025). Therapeutic effects and mechanisms of Xinmaitong formula for type 2 diabetes mellitus via GLP-1R signaling. *Front. Pharmacol.* 16:1575450. 10.3389/fphar.2025.1575450 40271065 PMC12014693

[B32] Qu Y. Li J. Qin Q. Wang D. Zhao J. An K. (2023). A systematic review and meta-analysis of inflammatory biomarkers in Parkinson’s disease. *NPJ Parkinsons Dis.* 9:18. 10.1038/s41531-023-00449-5 36739284 PMC9899271

[B33] Reynolds A. D. Banerjee R. Liu J. Gendelman H. E. Mosley R. L. (2007). Neuroprotective activities of CD4^+^CD25+ regulatory T cells in an animal model of Parkinson’s disease. *J. Leukoc. Biol.* 82 1083–1094. 10.1189/jlb.0507296 17675560

[B34] Roodveldt C. Bernardino L. Oztop-Cakmak O. Dragic M. Fladmark K. E. Ertan S. (2024). The immune system in Parkinson’s disease: What we know so far. *Brain* 147 3306–3324. 10.1093/brain/awae177 38833182 PMC11449148

[B35] Shi X. Huang H. Zhou M. Liu Y. Wu H. Dai M. (2021). Paeonol attenuated vascular fibrosis through regulating Treg/Th17 balance in a gut microbiota-dependent manner. *Front. Pharmacol.* 12:765482. 10.3389/fphar.2021.765482 34880759 PMC8646048

[B36] Shi Y. Wei B. Li L. Wang B. Sun M. (2022). Th17 cells and inflammation in neurological disorders: Possible mechanisms of action. *Front. Immunol.* 13:932152. 10.3389/fimmu.2022.932152 35935951 PMC9353135

[B37] Sommer A. Marxreiter F. Krach F. Fadler T. Grosch J. Maroni M. (2019). Th17 lymphocytes induce neuronal cell death in a human iPSC-based model of Parkinson’s disease. *Cell Stem Cell* 24:1006. 10.1016/j.stem.2019.04.019 31173705

[B38] Su D. Cui Y. He C. Yin P. Bai R. Zhu J. (2025). Projections for prevalence of Parkinson’s disease and its driving factors in 195 countries and territories to 2050: Modelling study of global burden of disease study 2021. *BMJ* 388:e080952. 10.1136/bmj-2024-080952 40044233 PMC11881235

[B39] Sun R. X. Guo Y. (2025). Gene signatures and immune correlations in Parkinson’s disease Braak stages. *Eur. J. Med. Res.* 30:278. 10.1186/s40001-025-02554-y 40229859 PMC11998164

[B40] Sun W. Wei J. Lin Z. Jiang C. Ping Y. Lu G. (2026). An involvement of ribonuclease L in Parkinson’s disease via modulating the Th17/Treg balance by microRNA-7. *Exp. Neurol.* 396:115517. 10.1016/j.expneurol.2025.115517 41135689

[B41] Sun X. Gu R. Bai J. (2024). Differentiation and regulation of CD4^+^ T cell subsets in Parkinson’s disease. *Cell. Mol. Life Sci.* 81:352. 10.1007/s00018-024-05402-0 39153043 PMC11335276

[B42] Thi Lai T. Kim Y. E. Nguyen L. T. N. Thi Nguyen T. Kwak I. H. Richter F. (2024). Microglial inhibition alleviates alpha-synuclein propagation and neurodegeneration in Parkinson’s disease mouse model. *NPJ Parkinsons Dis.* 10:32. 10.1038/s41531-024-00640-2 38302446 PMC10834509

[B43] Tian N. Yang C. Du Y. Chen M. Li B. Li D. (2025). Cannabinoid receptor 2 selective agonist ameliorates adjuvant-induced arthritis by modulating the balance between Treg and Th17 cells. *Front. Pharmacol.* 16:1532518. 10.3389/fphar.2025.1532518 39959429 PMC11825454

[B44] Wang C. Liao M. J. Wu Y. Lin H. Ye Z. Z. Ma W. Z. (2025). Pharmacological mechanisms of traditional Chinese medicine metabolites in regulating Treg cells: An integrative pathway review. *Front. Pharmacol.* 16:1527421. 10.3389/fphar.2025.1527421 41458964 PMC12738957

[B45] Wang L. Liang Y. Zhao C. Ma P. Zeng S. Ju D. (2025). Regulatory T cells in homeostasis and disease: Molecular mechanisms and therapeutic potential. *Signal Transduct. Target. Ther.* 10:345. 10.1038/s41392-025-02326-4 41087343 PMC12521743

[B46] Xie F. Shen B. Luo Y. Zhou H. Xie Z. Zhu S. (2024). Repetitive transcranial magnetic stimulation alleviates motor impairment in Parkinson’s disease: Association with peripheral inflammatory regulatory T-cells and SYT6. *Mol. Neurodegener.* 19:80. 10.1186/s13024-024-00770-4 39456006 PMC11515224

[B47] Xu Q. Qin W. Wu F. Z. Lin Y. Hong L. T. Chen D. (2021). Effect of roucongrong (Herba Cistanches Deserticolae) decoction on the substantia nigra through Wnt/β-catenin signaling pathway in rats with Parkinson’s disease induced by 6-hydroxydopamine hydrochloride. *J. Tradit. Chin. Med.* 41 762–770. 10.19852/j.cnki.jtcm.2021.05.010 34708635

[B48] Xu Lou I. Zhou H. Wan H. (2025). The critical role of Th17 cells and IL-17A in autoimmune and inflammation-associated neurological diseases: Mechanisms and therapeutic perspectives. *Front. Immunol.* 16:1656422. 10.3389/fimmu.2025.1656422 41357230 PMC12676946

[B49] Yang H. Wang Y. Xu Y. Wang C. (2025). CX3CR1: A potential microglia-specific PET imaging target in Alzheimer’s and Parkinson’s diseases. *Front. Pharmacol.* 16:1678159. 10.3389/fphar.2025.1678159 41424797 PMC12711710

[B50] Yang K. Lv Z. Zhao W. Lai G. Zheng C. Qi F. (2024). The potential of natural products to inhibit abnormal aggregation of α-Synuclein in the treatment of Parkinson’s disease. *Front. Pharmacol.* 15:1468850. 10.3389/fphar.2024.1468850 39508052 PMC11537895

[B51] Zeng J. Gao W. W. Yang H. Wang Y. N. Mei Y. Liu T. T. (2024). Sodium tanshinone IIA sulfonate suppresses microglia polarization and neuroinflammation possibly via regulating miR-125b-5p/STAT3 axis to ameliorate neuropathic pain. *Eur. J. Pharmacol.* 972:176523. 10.1016/j.ejphar.2024.176523 38552937

[B52] Zhang C. Huang P. Cheng H. Yuan X. Zhou M. Liu Y. (2025). Congrong Shujing Granules ameliorates mitochondrial associated membranes to against MPP+-induced neurological damage in the cellular model of Parkinson’s disease. *Front. Pharmacol.* 16:1509317. 10.3389/fphar.2025.1509317 40520174 PMC12162334

[B53] Zhang Y. Wang Z. Z. Sun H. M. Li P. Li Y. F. Chen N. H. (2014). Systematic review of traditional Chinese medicine for depression in Parkinson’s disease. *Am. J. Chin. Med.* 42 1035–1051. 10.1142/S0192415X14500657 25183301

[B54] Zhao M. Peng N. Zhou Y. Qu Y. Cao M. Zou Q. (2025). The immunoregulatory effects of total glucosides of peony in autoimmune diseases. *J. Leukoc. Biol.* 117:qiae095. 10.1093/jleuko/qiae095 38626175

[B55] Zheng K. Jia J. Yan S. Shen H. Zhu P. Yu J. (2020). Paeoniflorin ameliorates ulcerative colitis by modulating the dendritic cell-mediated TH17/Treg balance. *Inflammopharmacology* 28 1705–1716. 10.1007/s10787-020-00722-6 32472435 PMC7572351

[B56] Zheng M. Y. Luo L. Z. (2025). The role of IL-17A in mediating inflammatory responses and progression of neurodegenerative diseases. *Int. J. Mol. Sci.* 26:2505. 10.3390/ijms26062505 40141149 PMC11941770

