# Supplementary material for: Systems-level molecular and immunological evidence identifies Th17/Treg modulation as a key mechanism of CRSJ’s neuroprotection in Parkinson’s disease
Source: Front Aging Neurosci. 2026 Feb 18;18:1764634. doi: 10.3389/fnagi.2026.1764634 (PMC12957185; doi:10.3389/fnagi.2026.1764634)
Supplement: Supplementary file 1 [file Data_Sheet_1.docx]

**The original transcriptome data has been uploaded to the SRA database**

[https://www.ncbi.nlm.nih.gov/sra/PRJNA1266174].

**Supplementary Table S1. Composition of CongRong ShuJing granule**

| **Compounds** | **PubChem CID** | **Molecular Formula** | **Molecular Weight** |
| --- | --- | --- | --- |
| Echinacoside | 5281771 | C35H46O20 | 786.7 g/mol |
| Paeoniflorin | 442534 | C23H28O11 | 480.5 g/mol |
| Salvianolic acid B | 6451084 | C36H30O16 | 718.6 g/mol |
| Acteoside | 5281800 | C29H36O15 | 624.6 g/mol |
| Tanshinone IIa | 164676 | C19H18O3 | 294.3 g/mol |

**Supplementary Table S2. Composition of CongRong ShuJing granule**

| **Chinese name** | **Dose** | **Latin name** | **Familia** | **Region** |
| --- | --- | --- | --- | --- |
| Rou Cong Rong | 6 | Cistanche deserticola Ma | Orobanchaceae | Stem |
| Huang Jing | 12 | Polygonatum sibiricum | Liliaceae | Root |
| Dan Shen | 15 | Salvia miltiorrhiza Bunge | Lamiaceae | Root |
| Chi Shao | 12 | Radix Paeoniae Rubra | Ranunculaceae | Root |
| Mu Dan Pi | 10 | Cortex Moutan | Ranunculaceae | Root bark |

**Note:** All herbal ingredients were sourced from authenticated regions and verified according to the standards of the Chinese Pharmacopoeia (2020 Edition). The decoction process followed traditional formulation protocols under GMP conditions.

**Supplementary Table S3. Behavioral Scoring Criteria for PD Mouse Model**

| **Score** | **Behavioral Manifestations** |
| --- | --- |
| 0 | Normal posture and locomotion; no observable deficits. |
| 1 | Slight tremor or reduced spontaneous activity; normal righting reflex. |
| 2 | Moderate tremor, bradykinesia, or mild gait disturbance; capable of self-care. |
| 3 | Severe tremor and marked bradykinesia; difficulty in posture maintenance; slow response. |
| 4 | Loss of righting reflex, sustained akinesia; requires external stimulation for movement. |

**Note:** Behavioral scoring was conducted by two independent investigators blinded to treatment groups. Final scores represent the average of three consecutive daily assessments.

**Supplementary Table S4. Scoring Criteria for the Hanging Wire Test**

| **Behavioral Criteria** | **Score** |
| --- | --- |
| Mouse grips the wire securely with both hind limbs | 0 |
| Mouse grips the wire with only one hind limb | 0.5 |
| Mouse fails to grip the wire with either hind limb | 1.5 |
| Mouse immediately falls from the wire | 2 |

**Note:** Higher scores indicate greater motor impairment. Each mouse was tested three times, and the average score was used for analysis.

**Supplementary Table S5. Scoring Criteria for the Pole Test**

| **Time Taken to Descend the Pole** | **Score** |
| --- | --- |
| ≤ 4.00 seconds | 5 |
| 4.01–8.00 seconds | 4 |
| 8.01–12.00 seconds | 3 |
| 12.01–30.00 seconds | 2 |
| > 30.00 seconds | 1 |

**Note:** The pole test evaluates bradykinesia in mice. Higher scores represent better motor coordination and agility.

**Supplemental Table S6. List of Western Blot antibody**

| **Western Blot** | | | | | |
| --- | --- | --- | --- | --- | --- |
| **Target** | **Company** | **Cat** | **formula weight (KD)** | **Source of the antibody** | **Dilution** |
| ACTIN | Servicebio | GB15003 | 42 | Rabbit | 1:5000 |
| TGF-β | Servicebio | GB111876 | 44-55 | Rabbit | 1:1000 |
| smad3 | Servicebio | GB150085 | 55 | Rabbit | 1:1000 |
| RORG | Affinity | DF3196 | 58 | Rabbit | 1:1000 |
| a-syn | Servicebio | GB11404 | 19 | Rabbit | 1:1000 |
| CX3CR1 | Servicebio | GB11711 | 40 | Rabbit | 1:5000 |
| IL-17A | Servicebio | GB11110-1 | 17 | Rabbit | 1:1000 |
| Foxp3 | Servicebio | GB115746 | 47 | Rabbit | 1:1000 |
| iNOS | Servicebio | GB153965 | 131 | Rabbit | 1:1000 |
| Arg1 | Servicebio | GB11285 | 35 | Rabbit | 1:1000 |
| HRP-Sheep anti Rabbit IgG | Servicebio | GB23303 | - | Goat | 1:5000 |
| HRP-Sheep anti Rabbit IgG | Servicebio | GB23301 | - | Goat | 1:5000 |

**Supplemental Table S7. List of Flow Cytometry**

| **Flow Cytometry** | | | |
| --- | --- | --- | --- |
| **Target** | **Company** | **Cat** | **Dilution** |
| FITC Anti-Mouse CD4 Antibody[RM4-5] | elabscience | E-AB-F1353C | 1:400 |
| APC Anti-Mouse IL-17A Antibody | elabscience | E-AB-F1199E | 1:400 |
| APC Anti-Mouse CD25 Antibody | elabscience | E-AB-F1102E | 1:400 |
| PE Anti-Mouse Foxp3 Antibody | elabscience | E-AB-F1238D | 1:400 |
| Cell factor activation and protein blocking kit | elabscience | E-CK-A091 | - |
| Transcription Factor Staining Kit | elabscience | E-CK-A108 | - |
| FoxP3/Transcription Factor Staining Buffer Kit | LianKe | No. IC001) | - |

**Supplemental Table S8 List of immunohistology**

| **Immunofluorescence and Immunohistochemistry** | | | | |
| --- | --- | --- | --- | --- |
| **Target** | **Company** | **Cat** | **Dilution** | **Source of the antibody** |
| IL-17A | Servicebio | GB11110-1 | 1:400 | Rabbit |
| Foxp3 | Servicebio | No. GB112325 | 1:400 | Rabbit |
| RORγt | Affinity | DF3196 | 1:400 | Rabbit |
| CX3CL1 | proteintech | 60339-1-Ig | 1:400 | mouse |
| IBA1 | Servicebio | GB12105 | 1:400 | mouse |
| CD206 | Servicebio | GB113497 | 1:400 | mouse |
| CD86 | Servicebio | GB150054 | 1:400 | mouse |
| tyrosine hydroxylase | Proteintech | 25859-1-AP | 1:400 | Rabbit |
| Cy5-goat anti-mouse IgG | Servicebio | GB27301 | 1:200 | Goat |
| Alexa Fluor 594-goat anti rabbit IgG | Servicebio | GB28301 | 1:200 | Goat |
| FITC-goat anti rabbit IgG | Servicebio | GB22303 | 1:100 | Goat |
| Triton X-100 | Servicebio | No. G1204 | 1:100 | - |
| DAPI-containing mounting medium | Servicebio, | G1407 | - | - |
| biotin-labeled goat anti-mouse/rabbit IgG | Boster | SA1020 | - | - |
| DAB colorimetric kit | Boster | AR1022 | - | - |

**Supplementary Table S9 Molecular docking summary table**

| **Target** | **Kcal/mol** | **key hydrogen bond residue** | **major hydrophobic residue** | **Other important interactions** | **feature combination summarization** |
| --- | --- | --- | --- | --- | --- |
| Foxp3 | −6.6 | Phe340  Trp366 | Ile128, Trp348 | π–π：Phe373；carbon-hydrogen bond: Glu352；  vdW：Tyr342, Pro354, Met370 etc | Stable binding, multiple types of interactions jointly maintain pocket structure |
| RORγt | −8.6 | LEU472  ARG430  PRO468  PRO467 | LEU463 | vdW：LEU466, GLY470, LYS471, VAL424 etc | The highest affinity is dominated by multiple hydrogen bonds |
| α-syn | −6.2 | THR44 | LYS45 | vdW：LYS43, THR43, THR44 etc | Moderate binding, dominated by hydrogen bonds and hydrophobic interactions |
